# Potential involvement of the bone marrow in experimental Graves’ disease and thyroid eye disease

**DOI:** 10.3389/fendo.2023.1252727

**Published:** 2023-09-22

**Authors:** Anne Gulbins, Mareike Horstmann, Simone Keitsch, Matthias Soddemann, Barbara Wilker, Gregory C. Wilson, Ryan Zeidan, Gary D. Hammer, Anke Daser, Nikolaos E. Bechrakis, Gina-Eva Görtz, Anja Eckstein

**Affiliations:** ^1^ Molecular Ophthalmology, Department of Ophthalmology, University Hospital Essen, University Duisburg-Essen, Essen, Germany; ^2^ Department of Molecular Biology, University of Duisburg-Essen, Essen, Germany; ^3^ Department of Surgery, College of Medicine, University of Cincinnati, Cincinnati, OH, United States; ^4^ Sling Therapeutics Inc., Ann Arbor, MI, United States; ^5^ Endocrine Oncology Program, University of Michigan, Ann Arbor, MI, United States; ^6^ Department of Oto-Rhino-Laryngology, Head and Neck Surgery, University Hospital Essen, University Duisburg-Essen, Essen, Germany; ^7^ Department of Ophthalmology, University Hospital Essen, University Duisburg-Essen, Essen, Germany

**Keywords:** bone marrow, linsitinib, autoimmunity, inflammation, small molecule IGF-1R antagonist GD: Graves’ disease, thyroid eye disease (TED)

## Abstract

**Introduction:**

Graves’ disease is an autoimmune disorder caused by auto-antibodies against the thyroid stimulating hormone receptor (TSHR). Overstimulation of the TSHR induces hyperthyroidism and thyroid eye disease (TED) as the most common extra thyroidal manifestation of Graves’ disease. In TED, the TSHR cross talks with the insulin-like growth factor 1 receptor (IGF-1R) in orbital fibroblasts leading to inflammation, deposition of hyaluronan and adipogenesis. The bone marrow may play an important role in autoimmune diseases, but its role in Graves’ disease and TED is unknown. Here, we investigated whether induction of experimental Graves’ disease and accompanying TED involves bone marrow activation and whether interference with IGF-1R signaling prevents this activation.

**Results:**

Immunization of mice with TSHR resulted in an increase the numbers of CD4-positive T-lymphocytes (p ≤0.0001), which was normalized by linsitinib (p = 0.0029), an increase of CD19-positive B-lymphocytes (p= 0.0018), which was unaffected by linsitinib and a decrease of GR1-positive cells (p= 0.0038), which was prevented by linsitinib (p= 0.0027). In addition, we observed an increase of Sca-1 positive hematopietic stem cells (p= 0.0007) and of stromal cell-derived factor 1 (SDF-1) (p ≤0.0001) after immunization with TSHR which was prevented by linsitinib (Sca-1: p= 0.0008, SDF-1: p ≤0.0001). TSHR-immunization also resulted in upregulation of CCL-5, IL-6 and osteopontin (all p ≤0.0001) and a concomitant decrease of the immune-inhibitory cytokines IL-10 (p= 0.0064) and PGE2 (p ≤0.0001) in the bone marrow (all p≤ 0.0001). Treatment with the IGF-1R antagonist linsitinib blocked these events (all p ≤0.0001). We further demonstrate a down-regulation of arginase-1 expression (p= 0.0005) in the bone marrow in TSHR immunized mice, with a concomitant increase of local arginine (p ≤0.0001). Linsitinib induces an upregulation of arginase-1 resulting in low arginase levels in the bone marrow. Reconstitution of arginine in bone marrow cells *in vitro* prevented immune-inhibition by linsitinib.

**Conclusion:**

Collectively, these data indicate that the bone marrow is activated in experimental Graves’ disease and TED, which is prevented by linsitinib. Linsitinib-mediated immune-inhibition is mediated, at least in part, by arginase-1 up-regulation, consumption of arginine and thereby immune inhibition.

## 1 Introduction

Graves’ disease (GD) is the most common cause for hyperthyroidism, typically presenting in patients between 40–60 years (1, 2). Females are predominantly affected with a 8:1 ratio in comparison to men (3).

GD is an autoimmune disease of the thyroid which is caused by autoantibodies against the thyroid stimulating hormone receptor (TSHR) (TSHR Ab = TRAb), leading to an overproduction of thyroid hormones, overstimulation of the thyroid gland and consecutive hyperthyroidism (4, 5). Thyroid eye disease (TED) is the most frequent extra-thyroidal manifestation of GD and occurs in 20-30% of patients suffering from GD (3, 6). Autoreactive CD4+ T-cells and autoantibodies bind to the TSHR on orbital fibroblasts (OF’s), one of the key players in the pathogenesis of TED, in the orbital tissue. This results in the activation of the OF’s, differentiation into adipocytes, myofibroblasts and production of glycosaminoglycans (7). Severity of the course of TED is associated with the level of TSHR autoantibodies (TSHR Ab= TRAb) (8).

Another important receptor in the pathogenesis of TED is the insulin-like growth factor 1 receptor (IGF-1R) which has come more into focus in mediating the disease progression and is known to be overexpressed by thyroid follicular cells and OF’s as well as immune cells such in patients suffering from Graves’ disease and TED (9–11). In response to binding of the TSHR-autoantibodies (TRAb) to the TSHR in OF’s, the TSHR and IGF-1R form a complex and thereby crosstalk (12, 13), one central pathogenetic factor contributing to TED progression and development of typical symptoms, such as exophthalmos, double vision, dry eyes and in serious cases compressive optic neuropathy (14). Local anatomical factors are also significant, such as the size and shape of the orbit, the muscle thickening, that has already occurred and the perfusion situation. It is thought that the crosstalk between the TSHR and IGF-1R may be a key mechanism in the development and progression of TED. Therefore, targeting this crosstalk might be a promising therapeutic approach to treat TED (15).. One of these candidate drugs is linsitinib.

Linsitinib inhibits the autophosphorylation of the IGF-1R and therefore blocks important downstream signaling pathways, such as AKT and ERK signaling (16).

We have recently demonstrated that linsitinib, also known as OSI-906, a selective small-molecule dual inhibitor of the insulin like growth factor 1 receptor (IGF-1R) and insulin receptor (IR) (17), has a marked beneficial effect on the outcome of experimental TED (18). Linsitinib blocked the development and progression of the local pathologies of GD and TED in an *early* and *late* phase of the autoimmune disorder in target tissues and also downregulated the inflammation in the orbital tissue, indicating the clinical significance of our findings.

Recently Shi et. al., documented in an experimental autoimmune encephalomyelitis (EAE), an established mouse model for multiple sclerosis, that autoreactive T-cells migrate into the bone marrow and stimulate hematopoietic stem and progenitor cells to differentiate towards myeloid lineages that mediate inflammatory brain injury in the model (19). The autoreactive T-cells migrate into the bone marrow via the CXCL12-CXCR4 axis, while increased myelopoiesis is mediated in a CCL5-CCR5 dependent manner (19). CXCR4 knock-out attenuated the migration of myeline reactive CD4+ T-cells into the bone marrow, resulting in a reduced increase of hematopoietic stem cell proliferation (HSC) and, thus, also myeloid cell expansion (19). In addition, autoreactive T-cells in EAE showed an upregulation of CCL5 in the bone marrow, but also in the spleen and lymph nodes (19). This goes in line with the finding, that genetic knockdown of CCL5 led to an attenuated myelopoiesis in the bone marrow, induced by autoreactive T-cells, and deletion of CCR5 in the bone marrow reduced the development of EAE (19).

Similar to multiple sclerosis, TED is mainly a T-cell-, especially a Th1 and TH17 mediated autoimmune disease (20, 21) and the pathologies in the orbit are driven by immune cell infiltration, mainly CD4+ T-cell and macrophage infiltration, into the orbital fat and muscles (22, 23). However, it is unknown whether the bone marrow is altered in TED and plays a role in the pathogenesis of TED.

Here, we investigated in an experimental murine model of Graves’ disease, whether autoimmune GD and TED associates with bone marrow activation and whether linsitinib, an oral small molecule inhibitor of the IGF-1R and insulin receptor (IR), interferes with this activation process. In particular, we investigated whether immunization with TSHR results in (i) a change of the cellular components of the bone marrow, (ii) a potential recruitment or efflux of immune cells and (iii) a proliferation of stem cells, (iv) a release of pro-inflammatory cytokines driving an immune response and (v) a reduction of anti-inflammatory cytokines and the expression of arginase, which would also drive an immune response. We tested whether linsitinib has an effect on these changes of the bone marrow upon immunization with TSHR.

## 2 Methods

### 2.1 Mice

Female BALB/c inbred mice were purchased from Envigo Netherlands GmbH and housed under specifically pathogen-free conditions as described before (24). Mice were immunized as described below at an age of 6 weeks. We employed three groups of mice (18), i.e. healthy control mice immunized with non-immunogenic ß-Gal (n=10), TSHR immunized mice (n=10) and TSHR immunized mice that were treated with linsitinib (n=10). All bone marrow studies displayed in panels 2-6 were performed with these groups. Thus, we obtained bone marrow samples from each 10 BALB/c mice that were (i) immunized with the A-subunit of the TSHR and not further treated, (ii) immunized and treated with linsitinib or that were (iii) control-immunized with non-immunogenic ß-Gal and not further treated. Development of TED and changes in the orbit in these mice has been previously reported (18). These studies demonstrated that 90% of the mice that were immunized with TSHR developed TED. Linsitinib reduced the incidence of TED and only 10% of the mice that were immunized + treated with linsitinib developed TED. Specifically from our 28 mice used in this study, we had 8 ß-Gal mice (all healthy, none of them developed TED), 10 TSHR immunized mice not treated with linsitinib (9/10 mice developed TED) and 10 TSHR immunized mice treated with linsitinib (1/10 mice developed TED) (18). We followed the mice for 12 weeks. These studies also demonstrated that inflammation in the thyroid, with influx of CD3+ T-cells and morphological changes in the thyroid towards hyperthyroidism in the early phase after immunization. In a later phase we observed a slight decrease of hyperthyroidism and T-cell infiltration. Regarding orbital pathology, we observed structural changes like the formation of brown adipose tissue at all investigated time points (18). These data establish the suitability of the model and allowed us to investigate whether TED associates with changes in the bone marrow.

In a 2^nd^ group, we treated eight weeks old male C57BL/6 mice with linsitinib and also isolated bone marrow cells from these mice. The data obtained with these mice are displayed in panel 7. We used two different mouse strains to confirm the principal findings in an independent mouse strain.

All animal procedures were approved by the North Rhine Westphalian State Agency for Nature, Environment and Consumer Protection (LANUV), Germany, or by the local IACUC, University of Cincinnati, Cincinnati, USA. All experiments were performed according to the FELASA regulations and ARRIVE guidelines.

### 2.2 Immunizations and linsitinib treatment

**Figure 1:**
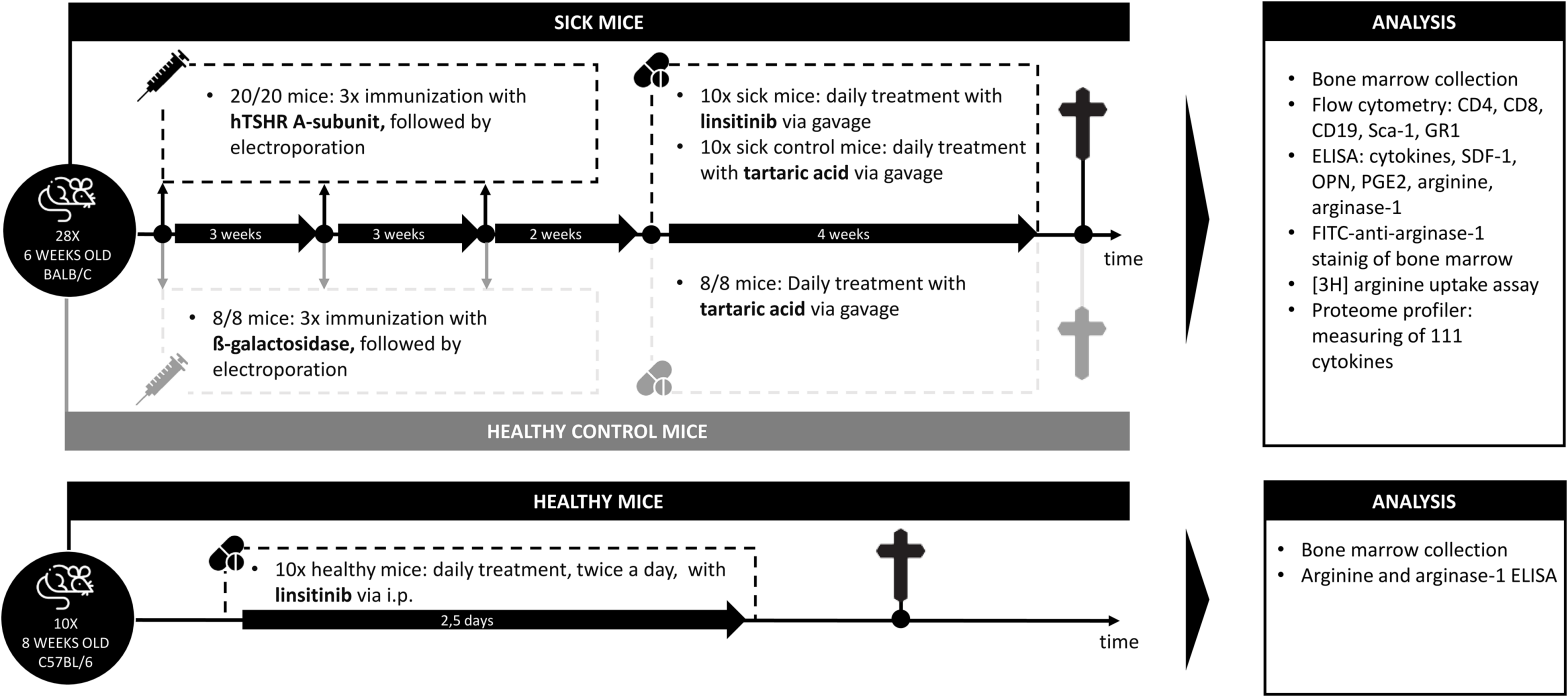
Outline of the induction of experimental Graves’ disease and treatment with linsitinib. Autoimmune hyperthyroidism and associated thyroid eye disease was induced by immunization of female BALB/c mice with a TSHR A-subunit encoding plasmid. The immunization procedure was repeated three times to obtain an optimal result. Treatment of THSR-immunized mice with linsitinib was initiated two weeks after the third immunization and performed for a total of four weeks. Untreated TSHR-immunized mice served as control group for the effect of treatment, while BALB/c mice that were immunized with a control plasmid encoding for non-immunogenic ß-Gal, were employed as healthy control mice (ß-Gal mice). Studies were performed six weeks after the last immunization.

We employed 10 animals/group. Specific immunization was obtained by immunizing mice with the eukaryotic expression plasmid pTriEx1.1Neo-human driving expression of the (h)TSHR A-subunit (also known as hTSHR289). Control mice were immunized with the pTri1Ex1.1Neo-ß-Gal plasmid, encoding non-immunogenic ß-Gal (named control ß-Gal group) as previously described (18). We immunized all mice three times with intervals of three weeks between the immunizations. TSHR immunized mice were split into two groups: One group was treated with linsitinib, while the other group was left untreated. All mice were sacrificed six weeks after the last immunization (see experimental outline in **Figure 1**). Thus, we followed the mice for 12 weeks totally.

None of the mice became thyrotoxic as determined by the already reported T4 levels in, the histology of the thyroid and the daily checked health status of these mice (18).

Linsitinib in the BALB/c mice was applied by daily gavage as previously described (18). The control mice (untreated or immunized but not treated with linsitinib) were also gavaged and the solvent was applied. For application of linsitinib in C57BL/6 mice please see below.

The exact timing of the experiment was chosen due to several other experiments our group performed with the BALB/C mice strain in order to induce Graves’ Disease and TED (25–27). In these experiments we identified that the autoimmune response starts to be present after the second immunization, which is why we this timing was chosen for treatment with linsitinib in the early group. The late group started after the third immunization, to represent the chronic state of the disease, since we previously showed, that the mice display features of a chronic inflammation at that time (25–27). This resamples two typical clinical situations: The early treatment group represents the group of patients who appear for therapy with a recently manifested active TED. The late treatment group represents the group of patients who appear for therapy with an already long-lasting chronic TED. We wanted to learn about the efficacy of linsitinib on both treatment conditions.

### 2.3 Bone marrow preparation and enzyme-linked immunosorbent assay

Briefly, the tibias and femurs of the mice were isolated, the muscle removed and the ends of the bones were cut off. The bone marrow was collected by rinsing the shaft with 2-3 ml PBS per bone using a 25 g needle. The bone marrow suspension was aliquoted and shock-frozen in liquid nitrogen. Samples were thawed, sonicated 3-times for 10 seconds each using a tip sonicator (Ultrasonic GE50) to achieve complete lysis and homogenization of the samples. The aliquots were analyzed using ELISA for cytokines (CCL5, SDF-1, IL-6, Osteopontin, IL-10, TNF-α, IL-17, IL-23), arginine, PGE_2_ and arginase-1 concentrations. ELISA assays were performed exactly following the instructions of the vendors. The mouse CCL-5 ELISA kit was from Abcam (# ab100739), mouse CCR5 ELISA from Aviva Systems Biology (# OKEH 03441), mouse Arginase-1 ELISA from Abcam (# ab269541), mouse SDF-1 ELISA from Abcam (# ab100741), mouse Osteopontin ELISA from Abcam (# ab100734), mouse IL-6 ELISA kit from R&D (# M6000B), mouse IL-1 ELISA from R&D (# MLB 00C), mouse IL-10 ELISA from R&D (# M1000B), mouse IL-17 ELISA from R&D (# M1700), mouse IL-23 ELISA from R&D (# M2300), mouse TNFα ELISA from R&D (# MTA 00B) and the PGE_2_ ELISA from R&D (# KGE004B). Arginine was quantified using a commercial assay from abcam (# ab241028). All values were normalized to protein in the samples.

### 2.4 Flow cytometry

In order to determine cell numbers in the bone marrow, around 100.000 bone marrow cells were freshly isolated. Aliquots of bone marrows were washed once in H/S (132 mM NaCl, 20 mM HEPES [pH 7.4], 5 mM KCl, 1 mM CaCl_2_, 0.7 mM MgCl_2_, 0.8 mM MgSO_4_), resuspended in 100 μL H/S, Fc-receptors were blocked by incubation for 30 min with Fc-block (anti-mouse CD16/CD32, Biolegend #101302), samples were washed once and aliquots were stained with FITC-coupled anti-mouse CD45 antibodies (1:200, clone RA3-6B2; Biolegend, #103228) and APC-coupled antibodies against mouse CD4 (1:200, clone GK1.5; Biolegend, #17-0041-81) or APC-coupled antibodies against mouse CD8 (1:200, clone 53-6.7; Biolegend, #17-0081-81) or PE-coupled antibodies against mouse CD19 (1:200, clone GD5; Biolegend, #115508) or APC-coupled antibodies against mouse Ly6G (Gr1) (1:200, clone RB6-BL5; Biolegend, #103107). In addition, cells were stained with Alexa 647-coupled anti-mouse CD45 antibodies (1:200, clone 30-F11; Biolegend, #103228) and FITC-coupled antibodies against mouse Sca-1 (1:200, clone D7; Biolegend, #108105). Cells were stained for 1 h at 4°C, washed once in H/S and analyzed on a FACS-Calibur. We analyzed the expression of the above described markers on 100 000 cells by flow cytometry.

### 2.5 Histopathology of bone marrow

Samples were fixed in 4% paraformaldehyde (Sigma), buffered with PBS to pH 7.2 – 7.4, for 48 h and the bones were de-calcified by incubation in 25% (w/v) EDTA (Roth, #6484.4) for 4 days. The EDTA solution was changed daily. Samples were then de-hydrated in an ethanol to xylol gradient series, paraffin embedded, sectioned at 6 μm and dewaxed, rehydrated, and treated with pepsin (Digest All; Invitrogen #1789292A) for 30 min. Sections were washed with water and PBS and blocked for 10 min at room temperature with PBS, and 5% fetal calf serum (FCS). The samples were then stained for arginase-1 employing a monoclonal mouse anti- murine arginase-1 antibody (clone GT5811; dilution 1:1000; Invitrogen, #MA 5-31577). Sections were incubated for 45 min in H/S at room temperature. Samples were then washed three times with PBS plus 0.05% Tween 20 and once with PBS and secondarily labeled with Cy3-coupled donkey anti-mouse F(ab)_2_ fragments (Jackson Immunoresearch, 715-166-150) in H/S plus 1% FCS for 45 min. Samples were washed again three times with PBS plus 0.05% Tween 20, once with PBS and were embedded in Mowiol. Samples were evaluated by confocal microscopy using a Leica TCS SL confocal fluorescence microscope.

Immunostainings were controlled with isotype control antibodies that showed no or very weak staining. These were mouse IgG2a isotype control antibodies (R&D, # MAB0031). We also included controls with secondary Cy3-coupled antibodies only.

### 2.6 Linsitinib injections and *in vitro* treatment with arginine in C57BL/6 mice

Eight weeks old, male, C57BL/6 mice were intraperitoneally (i.p.) injected with linsitinib at 10 mg/kg twice daily for 2.5 days (60 hrs), thus, in total 5 times. Linsitinib was dissolved in DMSO at 25 mg/mL and diluted into 250 mM sodiumacetate (pH 5.0). Mice were sacrificed 4 h after the last injection, the bone marrow was removed and cells were isolated by flushing the bones to obtain bone marrow as above. Aliquots were directly used to determine arginase-1 expression and arginine concentrations as above. In addition, cells were washed once in H/S and resuspended in 5% fetal calf serum in H/S. Fetal calf serum does not contain measurable concentrations of arginine, which was confirmed by using the arginine kit as above. Cells were then incubated for 24 h in H/S plus 5% FCS without arginine or supplemented with 25 μM or 100 μM arginine. Cells were then shock-frozen and concentrations of IL-10 and PGE_2_ were determined by ELISA as above.

### 2.7 Protein measurements

Protein concentrations in the samples were measured using the BioRad Protein Assay Dye (#500006) and served to normalize the samples. If cells were suspended in 5% FCS in H/S, the protein content of FCS was also determined and subtracted.

### 2.8 Arginine uptake

In order to determine the uptake of arginine into bone marrow cells, the cells were prepared as described above, resuspended in 5% fetal calf serum in H/S and incubated for 24 h with 10 μCi/mL [4,5-^3^H] arginine (specific activity 40 Ci/mmol; ARC, # ART0841). Cells were then extensively washed 4-times in H/S and the remaining intracellular radioactive arginine was quantified by liquid scintillation counting.

### 2.9 Quantification and statistical analysis

The manuscript includes all data, no animals were excluded and no data was excluded. Thus the data follows the intent-to-treat principle, although it is certainly not a clinical study.

Statistical analyses were performed using Prism 7 (GraphPad Software) and accomplished with One‐Way‐ANOVA for multiple comparisons between the different groups applying the Bonferroni correction for multiple testing. The *p* values for the pairwise comparisons were calculated after Bonferroni correction. P-values lower than 0.05 were considered as significant. In the ANOVA analysis we always compared the mean of each column with the mean of every other column and used the statistic program PRISM in order to calculate the adjusted p-values with the Bonferroni’s multiple comparison correction. The adjusted p-values are shown in the figures. The number of comparisons was 3 in all tests. The original p was set at 0.05, the corrected p is thus 0.05/3 = 0.0166. However, the computer program already adjusts the p-values.

All values were normally distributed, and the variances were similar. Statistical significance was set at a *p* value of 0.05 or lower (two-tailed). The sample size planning was based on the results of two-sided Wilcoxon-Mann-Whitney tests (free software: G*Power, Version 3.1.7, University of Duesseldorf, Germany). Investigators were blinded to results of histologic analyses and to animal identity. Before the experiments, animals were randomly assigned to cages by a technician who was not involved in the experiments. Cages were randomly assigned to the various experimental groups. Data is shown as arithmetic mean ± standard deviation (SD), and the exact p-values are shown in the figures. Changes between mouse groups with p‐values > 0.05 are regarded as not statistically significant are not shown in the graphs.

## 3 Results

**Figure 2:**
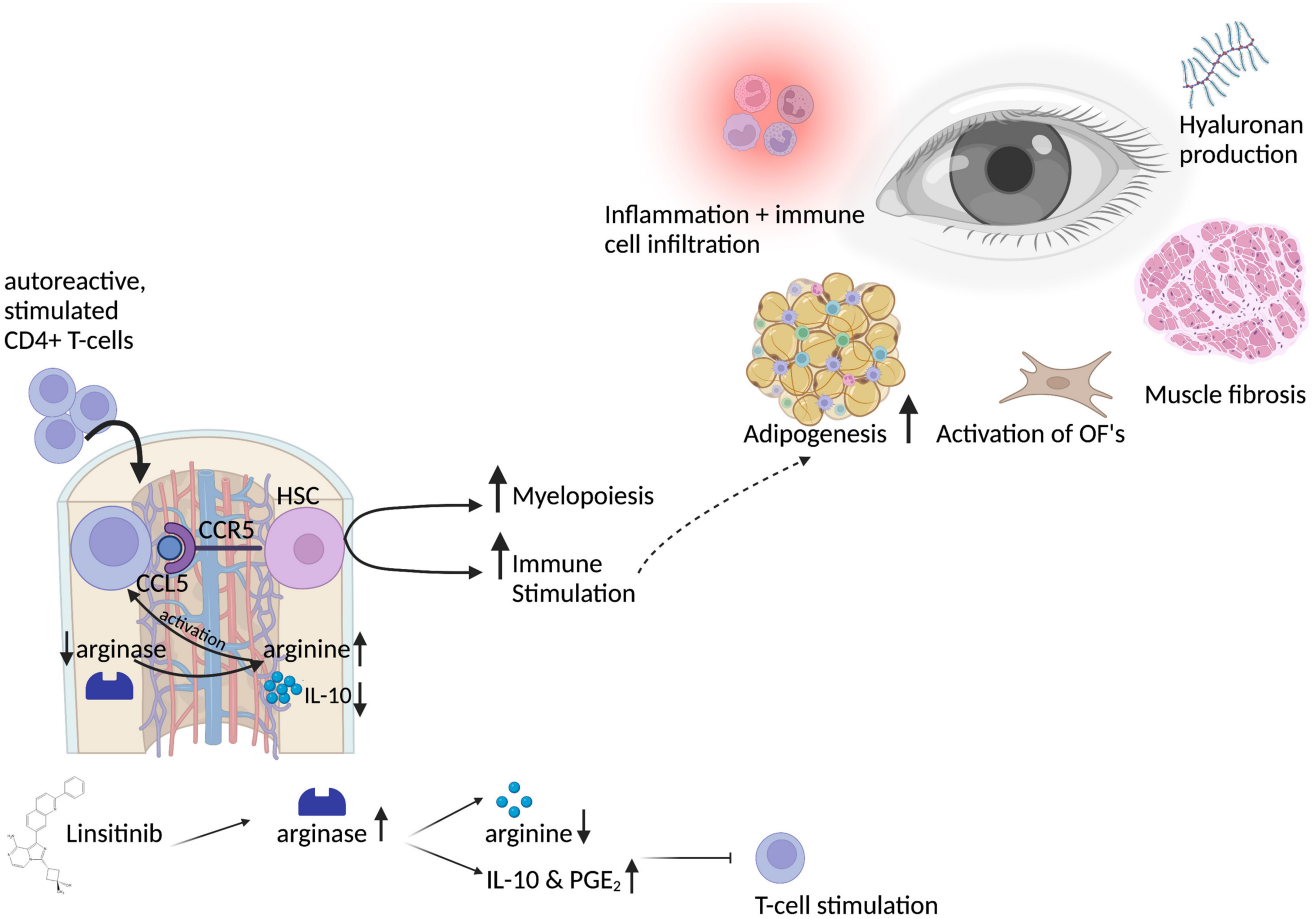
Graphical abstract of the bone marrow activation in thyroid eye disease and the role of linsitinib. Autoreactive CD4+ T-cells, stimulated by auto-antigens like the TSHR in thyroid eye disease, migrate into the bone marrow in a CCL5-CCR5 dependent manner. CCL5 stimulates and activates hematopoietic stem cells, which then proliferate, differentiate and drive myelopoiesis. Immune cells from the bone marrow might migrate to the periphery and to the eye in which they lead to inflammation and trigger the immune response. This leads to the typical symptoms of thyroid eye disease, like activation of orbital fibroblasts (OF), increased adipogenesis, production of hyaluronan acid and muscle fibrosis. Activation of CD4+ T-cells in the bone marrow requires L-arginine, an amino-acid which is pivotal for the activation, stimulation and proliferation of T-cells. Linsitinib, a small-molecule dual inhibitor of the insulin like growth factor 1 receptor (IGF-1R) and insulin receptor (IR) leads to upregulation of arginase-1 expression and the formation of IL-10 and PGE_2_, which results in the depletion of arginine and thereby inhibition of T-cell functions. Further, IL-10 and PGE_2_ promote an anti-inflammatory micromilieu, which inhibits the autoimmune response. Created with BioRender.com.

In the present study, we investigated the role of bone marrow cells during development of experimental Graves’ disease and TED and whether the bone marrow is involved in the immune inhibitory effect of the IGF-1R/IR antagonist linsitinib. The study design is outlined in **Figure 1**. In addition, an overview of the results is displayed in **Figure 2**.

### 3.1 CD4+ T-cell infiltration in the bone marrow upon immunization of mice with TSHR

**Figure 3:**
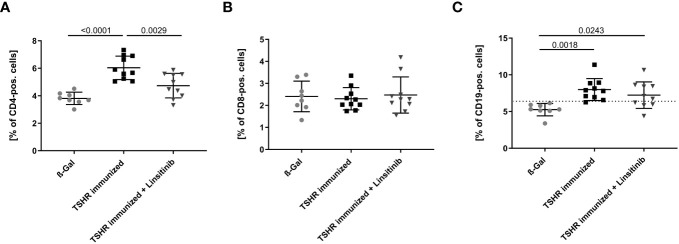
Effect of linsitinib treatment on T-cell and B-cell counts. Total counts of CD4+ **(A)**, CD8+ **(B)** T-cells and CD19+ B-cells **(C)** in bone marrow samples obtained from mice from each group, i.e., ß-Gal, TSHR immunized and TSHR immunized treated with linsitinib were determined by flow cytometry. Shown are the mean ± SD, n=8 for ß-Gal, n=10 for TSHR immunized and n=10 for TSHR immunized + Linsitinib. Statistical differences were determined using one-way-ANOVA; exact p-values are given in the figures. The upper 99% CI of the CD19+ B-cell count in the ß-Gal group is indicated by a dotted line.

We initially determined the effects of immunization of mice with the TSHR and treatment of TSHR-immunized mice with linsitinib on the counts of lymphocytes, i.e., T- and B-lymphocytes, in the bone marrow using flow cytometry. To this end, mice were immunized 3-times with TSHR or control vector plasmid (β-Gal). Immunized mice were left untreated or treated with linsitinib for 4 weeks starting 2 weeks after the 3^rd^ immunization (18). The bone marrow was then isolated at end of the linsitinib treatment. Our studies revealed a marked increase of CD4+ T-cells in the bone marrow (p ≤0.0001), upon immunization with the TSHR (**Figure 3A**). Linsitinib decreased and almost normalized the counts of CD4+ T-cells in the bone marrow of immunized mice (p = 0.0029). Counts of CD8+ T-cells were not altered in the bone marrow upon immunization of mice with the TSHR or treatment of TSHR immunized mice with linsitinib (**Figure 3B**), suggesting a specific involvement of CD4+ T-lymphocytes in the immune response of the bone marrow during TED. In addition to an increase of CD4+-T-cells in the bone marrow upon immunization, we also observed an increase of CD19+-B-lymphocytes after immunization (p= 0.0018) which was, however not affected by linsitinib (**Figure 3C**).

### 3.2 Effect of TSHR-immunization on stimulation of bone marrow stem cells

**Figure 4:**
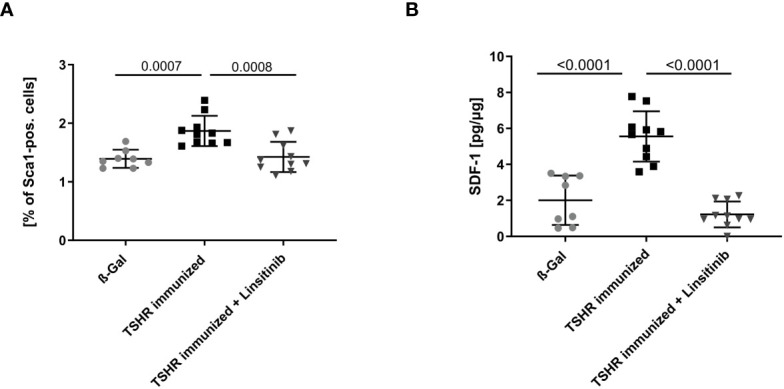
Inhibition of bone marrow activation upon treatment with linsitinib. Total counts of Sca-1+ **(A)** and concentrations of SDF-1 **(B)** in bone marrow samples obtained from mice from each group, i.e., ß-Gal, TSHR immunized and TSHR immunized treated with linsitinib were determined either by flow cytometry **(A)** or ELISA **(B)**. Shown are the mean ± SD, n=8 for ß-Gal, n=10 for TSHR immunized and n=10 for TSHR immunized + Linsitinib. Statistical differences were determined using one-way-ANOVA; exact p-values are given in the figures.

In order to further investigate stimulation of the bone marrow upon immunization with TSHR and the effects of linsitinib on this process, we determined the expression of Stem cell antigen-1 (Sca-1) by flow cytometry of freshly isolated bone marrow. Hematopoietic stem and progenitor cells (HSPCs) express the surface molecule Sca-1 (28) and, thus, it serves as a reproducible marker for stem cell activation. Mice were immunized and treated as above and the bone marrow was isolated, stained with fluorescent-labelled anti-Sca-1 antibodies and analyzed by flow cytometry. Immunization of mice with TSHR led to an increase of Sca-1 (p= 0.0007), which was completely prevented by treatment with linsitinib (**Figure 4A**), indicating increased hematopoiesis after immunization, which was completely prevented by treatment with linsitinib. The notion of stem cell activation upon TSHR immunization is supported by the finding that the bone marrow of immunized mice showed a significantly increased concentration (p ≤0.0001), of stromal cell-derived factor 1 (SDF-1), also known as CXCL12 (**Figure 4B**), which is involved in the control of homing and mobilization of bone marrow stem cells and inflammatory processes by initiating immune cells such as neutrophils, eosinophils, basophils, lymphocytes, and monocytes in response to inflammatory signals (29, 30). Linsitinib completely reversed the increase of SDF-1 in immunized mice (**Figure 4B**).

### 3.3 Effect of TSHR-immunization on myeloid cells in the bone marrow

**Figure 5:**
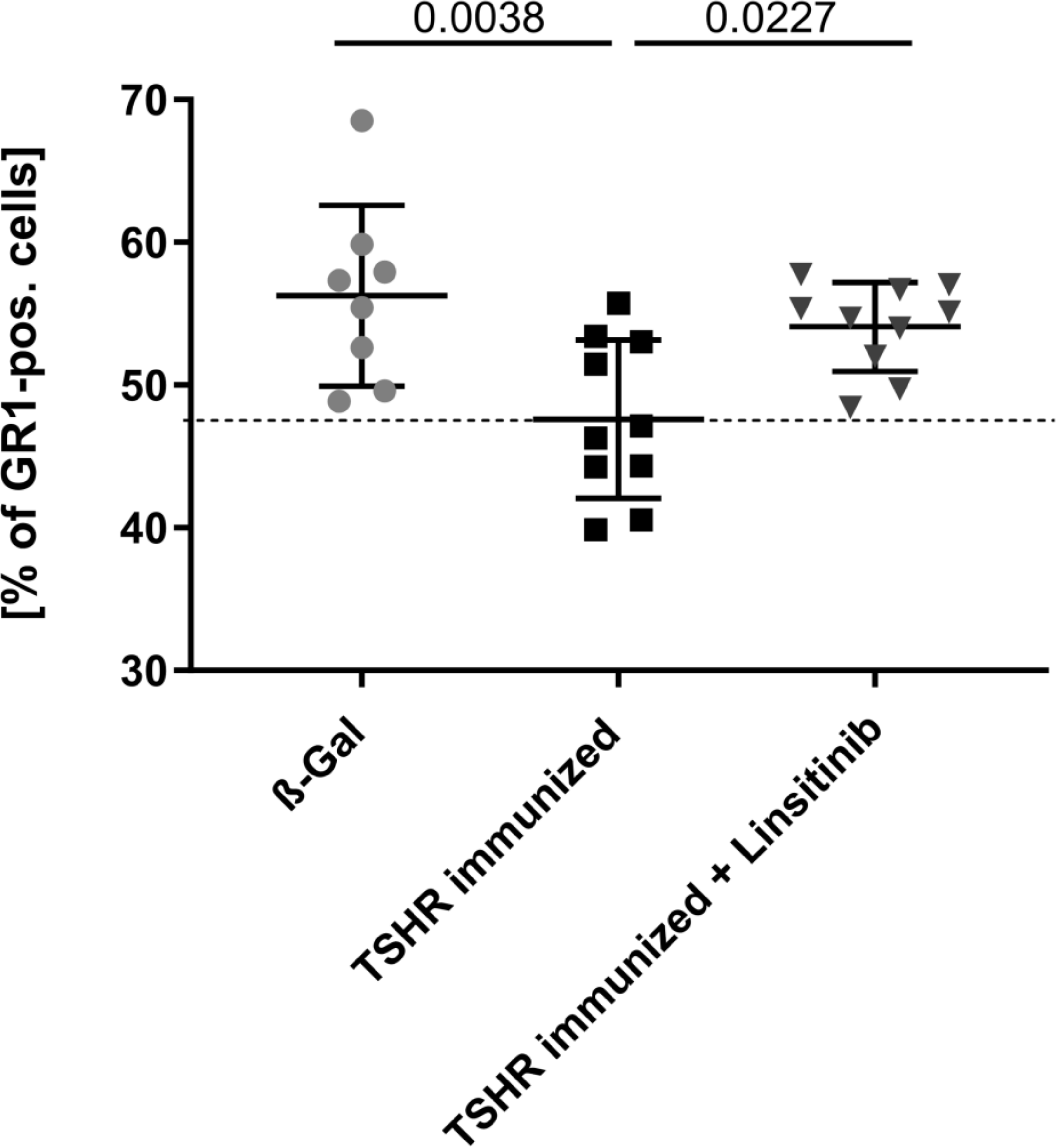
Linsitinib inhibits migration of myeloid cells from the bone marrow in the periphery. Counts of GR1 expressing cells in bone marrow samples obtained from mice from each group, i.e., ß-Gal, TSHR immunized and TSHR immunized treated with linsitinib were determined by flow cytometry. Shown are the mean ± SD, n=8 for ß-Gal, n=10 for TSHR immunized and n=10 for TSHR immunized + Linsitinib. Statistical differences were determined using one-way-ANOVA; exact p-values are given in the figures. The upper 99% CI of the GR1 cell count in the ß-Gal group is indicated by a dotted line.

To compare the number of myeloid cells in the bone marrow between the different groups, mice were treated and bone marrow isolated as above. Bone marrow cells were stained with fluorescently-labelled anti-GR1 antibodies and analyzed by flow cytometry. GR1, also known as Ly-6G, is a cell surface marker that is primarily expressed on myeloid cells (31). Immunization of mice with TSHR led to a decrease of GR1-positive cells in the bone marrow (p= 0.0038) indicating a mobilization of myeloid cells from the bone marrow to the periphery (**Figure 5**). These cells may migrate to the orbit and mediate disease progression in TED. Linsitinib significantly (p= 0.0027) prevented the reduction of the counts of GR1-positive cells in the bone marrow of immunized mice (**Figure 5**).

### 3.4 Effect of TSHR-immunization on cytokines in the bone marrow

**Figure 6:**
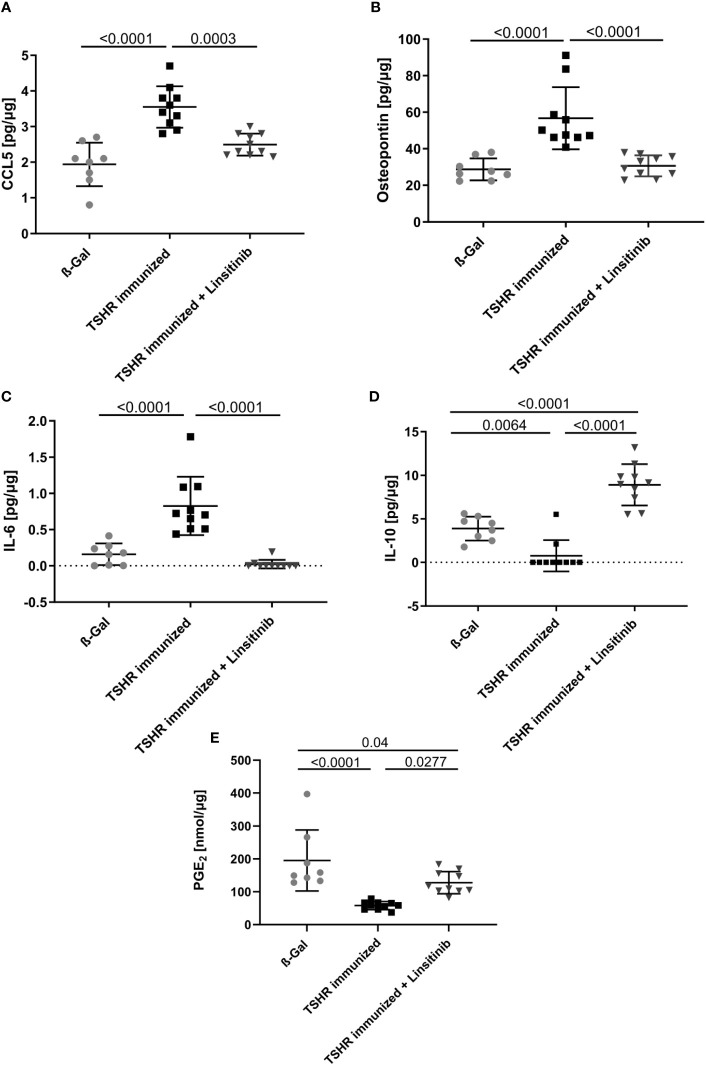
Reduction of proinflammatory cytokines and upregulation of anti-inflammatory cytokines in response to linsitinib treatment. Total concentrations of the proinflammatory cytokines, CCL5 **(A)**, Osteopontin **(B)** and IL-6 **(C)** and of the anti-inflammatory cytokines IL-10 **(D)** and PGE_2_ **(E)** in bone marrow samples obtained from mice from each group, i.e., ß-Gal, TSHR immunized and TSHR immunized treated with linsitinib were determined via ELISA. Shown are the mean ± SD, n=8 for ß-Gal, n=10 for TSHR immunized and n=10 for TSHR immunized + Linsitinib. Statistical differences were determined using one-way-ANOVA; exact p-values are given in the figures.

In order to show the activation of the bone marrow via the CCL5(Rantes)-CCR5 axis, which has been previously implied as a major pathway activating bone marrow by T-lymphocytes during autoimmune encephalitis (19), we performed an ELISA for CCL5. Our data indicate that immunization of mice with THSR significantly (p ≤0.0001) increased CCL5 concentrations in the bone marrow (**Figure 6A**). Linsitinib inhibited CCL5 upregulation and almost normalized the amount of CCL5 in the bone marrow (**Figure 6A**). To further characterize immune stimulation of the bone marrow, we performed an unbiased multiplex cytokine assay measuring expression levels of 111 cytokines (**Table 1**). The array studies showed an upregulation of CCL5, CD14, CD40, IL-33, LDL-R, Lipocalin-2 and Osteopontin in TSHR immunized mice in comparison to healthy ß-Gal mice and TSHR mice treated with linsitinib. We focused on paradigmatic pro-inflammatory cytokines, in particular Osteopontin, and measured the concentration of Osteopontin by quantitative ELISA. These studies indicate a 2-fold increase of Osteopontin in the bone marrow after immunization compared to controls (p ≤0.0001) (**Figure 6B**). The increase of Osteopontin in the bone marrow was normalized by treatment with linsitinib (**Figure 6B**). Osteopontin is known to be a multifunctional cytokine, expressed by T-cells, fibroblasts and other cells and for example stimulates T-cells and macrophages in inflammation processes (32–34). To confirm the effects of linsitinib on pro-inflammatory cytokines, we also determined the concentration of IL-6 in the bone marrow of immunized, immunized + linsitinib-treated and control mice. IL-6 is a known pro-inflammatory cytokine (35). The IL-6 concentration markedly increased in TSHR immunized mice (p ≤0.0001), which was completely abrogated by treatment with linsitinib (**Figure 6C**). We also determined the concentrations of typical cytokines involved in autoimmune disorders or inflammation, such as TNF-α, IL-1β, IL-17, IL-23 and CCR5 concentrations in the bone marrow prior and after immunization ± treatment with linsitinib, but the concentrations were either very low or undetectable (not shown).

**Table 1:** Regulation of cytokines in a proteome profiler.

Analyte	ß-Gal	TSHR	TSHR + linsitinib	Analyte	TSHR	TSHR + linsitinib
Adiponectin/Acrp30				Adiponectin/Acrp30		
Amphiregulin				Amphiregulin		
Angiopoietin-1				Angiopoietin-1		
Angiopoietin-2				Angiopoietin-2		
Angiopoietin-like 3				Angiopoietin-like 3		
BAFF/BlyS/TNFSF13B	↑↑	↑		BAFF/BlyS/TNFSF13B	↑	
C1qR1/CD93				C1qR1/CD93		
CCL2/JA/MCP-1				CCL2/JA/MCP-1		
CCL3/CCL4/MIP-1α/β				CCL3/CCL4/MIP-1α/β		
CCL5/Rantes	↑	↑↑	↑	CCL5/Rantes	↑↑	↑
CCL6/C10	↑↑	↑	↑	CCL6/C10	↑	↑
CCL11/Eotaxin				CCL11/Eotaxin		
CCL12/MCP-5				CCL12/MCP-5		
CCL17/TARC				CCL17/TARC		
CCL19/MIP-3β				CCL19/MIP-3β		
CCL20/MIP-3α				CCL20/MIP-3α		
CCL21/6Ckine				CCL21/6Ckine		
CCL22/MDC				CCL22/MDC		
CD14		↑		CD14	↑	
CD40/TNFRSF5	↑	↑↑	↑	CD40/TNFRSF5	↑↑	↑
CD160				CD160		
Chemerin				Chemerin		
Chitinase 3-like 1	↑↑↑↑	↑↑↑↑	↑↑↑↑	Chitinase 3-like 1	↑↑↑↑	↑↑↑↑
Coagulation Factor III/Tissue Factor				Coagulation Factor III/Tissue Factor		
Complement Component C5/C5a				Complement Component C5/C5a		
Complement Factor D				Complement Factor D		
C-reactive Protein/CRP				C-reactive Protein/CRP		
CX3CL1/Fractalkine				CX3CL1/Fractalkine		
CXCL1/KC				CXCL1/KC		
CXCL2/MIP-2				CXCL2/MIP-2		
CXCL9/MIG				CXCL9/MIG		
CXCL10/IP-10				CXCL10/IP-10		
CXCL11/I-TAC				CXCL11/I-TAC		
CXCL13/BLC/BCA-1				CXCL13/BLC/BCA-1		
CXCL16				CXCL16		
Cystatin C	↑↑	↑↑	↑	Cystatin C	↑↑	↑
DKK-1				DKK-1		
DPPIV/CD26				DPPIV/CD26		
EGF				EGF		
Endoglin/CD105				Endoglin/CD105		
Endostatin				Endostatin		
Fetuin A/AHSG	↑↑	↑↑	↑↑	Fetuin A/AHSG	↑↑	↑↑
FGF acidic				FGF acidic		
FGF-21				FGF-21		
Flt-3 Ligand				Flt-3 Ligand		
Gas 6				Gas 6		
G-CSF				G-CSF		
GDF-15				GDF-15		
GM-CSF				GM-CSF		
HGF				HGF		
ICAM-1/CD54				ICAM-1/CD54		
IFN-y				IFN-y		
IGFBP-1				IGFBP-1		
IGFBP-2				IGFBP-2		
IGFBP-3				IGFBP-3		
IGFBP-5				IGFBP-5		
IGFBP-6				IGFBP-6		
IL-1α/IL-1F1				IL-1α/IL-1F1		
IL-1β/IL-1F2				IL-1β/IL-1F2		
IL-1ra/IL-1F3				IL-1ra/IL-1F3		
IL-2				IL-2		
IL-3				IL-3		
IL-4				IL-4		
IL-5				IL-5		
IL-6				IL-6		
IL-7				IL-7		
IL-10				IL-10		
IL-11				IL-11		
IL-12 p40				IL-12 p40		
IL-13				IL-13		
IL-15				IL-15		
IL-17A				IL-17A		
Il-22				Il-22		
Il-23				Il-23		
Il-27 p28				Il-27 p28		
IL-28A/B				IL-28A/B		
IL-33	↑	↑↑	↑	IL-33	↑↑	↑
LDL R	↑	↑↑	↑	LDL R	↑↑	↑
Leptin				Leptin		
LIF				LIF		
Lipocalin-2/NGAL	↑↑	↑↑↑	↑↑	Lipocalin-2/NGAL	↑↑↑	↑↑
LIX	↑↑↑↑	↑↑↑↑	↑↑↑↑	LIX	↑↑↑↑	↑↑↑↑
M-CSF				M-CSF		
MMP-2				MMP-2		
MMP-3				MMP-3		
MMP-9	↑↑↑↑	↑↑↑↑	↑↑↑↑	MMP-9	↑↑↑↑	↑↑↑↑
Myeloperoxidase	↑↑↑	↑↑↑	↑↑↑	Myeloperoxidase	↑↑↑	↑↑↑
Osteopontin (OPN)	↑	↑↑	↑	Osteopontin (OPN)	↑↑	↑
Osteoprotegerin/TNFRSF11B				Osteoprotegerin/TNFRSF11B		
PD-ECGF/Thymidine phosphorylase				PD-ECGF/Thymidine phosphorylase		
PDGF-BB				PDGF-BB		
Pentraxin 2/SAP				Pentraxin 2/SAP		
Pentraxin 3/TSG-14	↑↑↑	↑↑↑	↑↑↑	Pentraxin 3/TSG-14	↑↑↑	↑↑↑
Periostin/OSF-2				Periostin/OSF-2		
Pref-1/DLK-1/FA1				Pref-1/DLK-1/FA1		
Proliferin				Proliferin		
Proprotein Convertase 9/PCSK9				Proprotein Convertase 9/PCSK9		
RAGE				RAGE		
RBP4	↑↑	↑↑	↑↑	RBP4	↑↑	↑↑
Reg3G				Reg3G		
Resistin				Resistin		
E-Selectin/CD62E				E-Selectin/CD62E		
P-Selectin/CD62P	↑↑	↑↑	↑↑	P-Selectin/CD62P	↑↑	↑↑
Serpin E1/PAI-1				Serpin E1/PAI-1		
Serpin F1/PEDF				Serpin F1/PEDF		
Thrombopoietin				Thrombopoietin		
TIM-1/KIM-1/HAVCR				TIM-1/KIM-1/HAVCR		
TNF-α				TNF-α		
VCAM-1/CD106				VCAM-1/CD106		
VEGF				VEGF		
WISP-1/CCN4				WISP-1/CCN4		
positive control (A1/A2, A23/A24, J1/J2)	↑↑↑↑	↑↑↑↑	↑↑↑↑			
negative control (J23/J24)						

Unbiased analysis of 111 cytokines in bone marrow samples from each group, i.e., ß-Gal, TSHR immunized and TSHR immunized treated with linsitinib, reveals an upregulation of several pro-inflammatory cytokines upon immunization and their down-regulation upon treatment with linsitinib. Intensity of the analytes were compared to the positive control, with ↑↑↑↑ for the same intensity as the positive control, ↑↑↑ for a slight less intense analyte, ↑↑ for half the intensity compared to the positive control and ↑ for a relatively low intensity, but still more than the negative control. For transparency, the foil with the analytes for each group; ß-Gal, TSHR and TSHR + linsitinib is shown as well. The yellow shadings indicate that we saw a difference in the cytokines between the TSHR immunized group and the TSHR immunized group treated with linsitinib, so an increase in the TSHR immunized group and a decrease in the TSHR immunized group treated with linsitinib.

In contrast to CCL-5 and the cytokines IL-6 and Osteopontin, which drive different aspects of the immune response, IL-10 and PGE_2_ serve to down-regulate immune responses with potent anti-inflammatory characteristics (36, 37). The concentrations of IL-10 and PGE_2_ in the bone marrow decreased after immunization of mice with TSHR (IL-10: p= 0.0064, PGE_2_: p ≤0.0001) (**Figures 6D, E**). Linsitinib increased the concentration of IL-10 and PGE_2_ in the bone marrow of immunized mice (**Figures 6D, E**).

### 3.5 Effects of TSHR immunization on arginase-1 in the bone marrow

**Figure 7:**
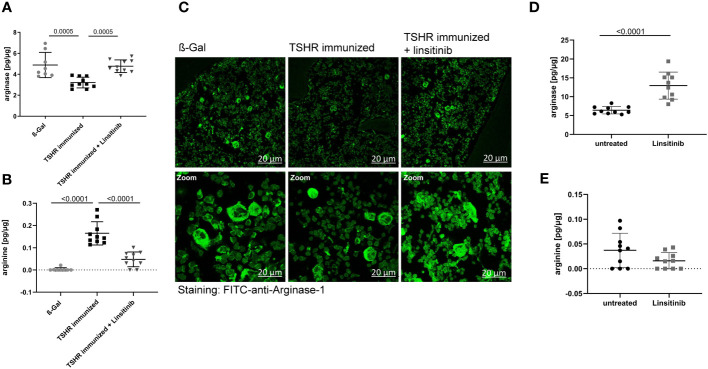
Linsitinib inhibits T-cell activation by inducing arginase-1 expression in the bone marrow. **(A, B)** Total arginase-1 and arginine concentrations in bone marrow samples obtained from mice from each group, i.e., ß-Gal, TSHR immunized and TSHR immunized treated with linsitinib were determined via ELISA. **(C)** Bone marrow samples were fixed, paraffin embedded and sections were stained for arginase-1 using FITC-coupled anti-arginase-1 antibodies. Samples were analyzed by confocal microscopy, magnification was 400-fold **(D, E)** In order to further analyze the effect of linsitinib on arginase-1 and arginine, mice were injected 5-times over 2.5 days with linsitinib (10 mg/kg, twice daily i.p.), bone marrow cells were isolated 4 h after the last injection and the expression of arginase-1 **(D)** and arginine levels **(E)** in the bone marrow were determined using ELISA. **(A, B, D, E)** Show the mean ± SD, n=8 for ß-Gal, n= 10 for TSHR immunized, n=10 for TSHR immunized + Linsitinib and n= 10 for solvent-treated (untreated) mice or n=10 for linsitinib treated mice. Statistical differences were determined using one-way-ANOVA **(A, B)** or an unpaired, t-test **(D, E)**, exact p-values are given in the figures. **(C)** shows representative images of the bone marrow of each group analyzed in panel **(A)**. At least 3 sections per group were analyzed, thus a total of at least 21 sections was analyzed per group.

Arginase-1 is an enzyme which metabolizes L-arginine to L-ornithine and urea in the urea cycle (38, 39). It has been shown that T-cell proliferation and activation is highly dependent on arginine and thus arginase-1 activity (40). T-cells require arginine to proliferate properly and mediate the immune response. Arginase-1 inhibitors, which consequently increase the arginine availability for T-cells, not only activated immune cells, but also led to a strong hyperactivation of CD4+ and CD8+ T-cells (40).. Therefore, we investigated the role of arginase-1 in TED and the effects of linsitinib on arginase-1. Immunization with TSHR led to a decrease of arginase-1 expression in the bone marrow (**Figure 7A**) and a concomitant increase of arginine concentrations (**Figure 7B**). Treatment with linsitinib completely normalized arginase-1 expression levels in the bone marrow of immunized mice (p= 0.0005) (**Figure 7A**) and reduced arginine concentrations to baseline(p ≤0.0001) (**Figure 7B**).

These data suggest that linsitinib inhibits T-cell activation by inducing the expression of arginase-1 in the bone marrow thereby inducing an immunosuppressive micromilieu. This notion was supported by histology upon staining of bone marrow sections with FITC-coupled anti-arginase-1 antibodies. TSHR immunized mice showed significantly lower numbers of arginase-1-positive cells in the bone marrow, which was corrected to normal levels by treatment of immunized mice with linsitinib (**Figure 7C**).

To further investigate the effects of linsitinib on arginase-1 and the significance for immune cell stimulation, we treated 10 C57BL/6 mice for 2.5 day with i.p. injections (10 mg/kg) of linsitinib, twice daily. Control mice were injected with solvent. We then isolated bone marrow cells 4h after the last injection and determined arginase-1 expression and arginine concentrations in these samples. The results demonstrate that linsitinib induced expression of arginase-1 in the bone marrow (p ≤0.0001) (**Figure 7D**) and slightly reduced the anyway low basal arginine levels in the bone marrow of these non-immunized mice (**Figure 7E**).

### 3.6 Effects of linsitinib on arginase

**Figure 8:**
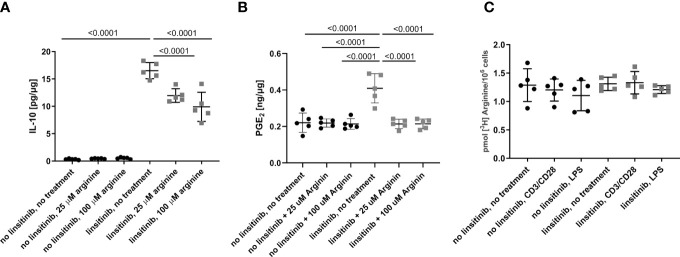
Linsitinib regulates the immune response via IL-10 production in the bone marrow. **(A, B)** C57BL/6 mice were treated for 2.5 day with i.p. injections (10 mg/kg) of linsitinib or the solvent, twice daily. Bone marrow were isolated and cultured in the absence of arginine to mimic high expression of arginase-1 or in presence of 25 μM and 100 μM arginine to mimic a down-regulation of arginase-1. IL-10 **(A)** and PGE_2_ **(B)** concentrations in bone marrow cells from mice of each group, i.e treated with linsitinib or solvent, respectively, and either left untreated *in vitro*, treated with 25 μM arginine and 100 μM arginine were determined by ELISA. **(C)** The uptake of [^3^H] arginine was measured over 24 h in the bone marrow of either untreated and unstimulated cells, cells stimulated with CD3/CD28, cells stimulated with LPS, cells treated with linsitinib but left untreated, cells treated with linsitinib and stimulated with CD3/CD38 and cells treated with linsitinib and stimulated with LPS. Shown are the mean ± SD, n=5, Statistical differences were determined using one-way-ANOVA; exact p-values are given in the figures.

To define the role of arginase-1 in linsitinib-mediated immune suppression, we injected mice with linsitinib as above, isolated bone marrow cells and cultured the cells in the absence or presence of arginine for 24 h *in vitro*. We then determined IL-10 and PGE_2_ concentrations. Linsitinib induced an increase of IL-10 and PGE_2_ concentrations in the bone marrow cells (p ≤0.0001) compared to untreated to cells in the medium lacking arginine, which mimics a high activity of arginase-1 with depletion of arginine *in vivo* (**Figures 8A, B**). Reconstitution of arginine concentration dose-dependently suppressed, at least partly, linsitinib- induced IL-10 and PGE_2_ expression in bone marrow cells (**Figures 8A, B**), indicating that linsitinib mediates immune suppression, at least in part, by upregulation of arginase-1 and concomitant depletion of arginine.

This phenotype could also be explained by an inhibition of arginine uptake by linsitinib, since arginine is transported into the cells via plasma membrane transporters (41–43) and inhibition of this uptake could also decrease the arginine availability for the cells in an arginase-1-independent manner. Therefore, we determined arginine uptake in the bone marrow of either untreated and unstimulated cells, cells stimulated with CD3/CD28 in order to induce T-cell stimulation, cells stimulated with lipopolysaccharide (LPS) to induce myeloid cell activation, cells treated with linsitinib but left untreated, cells treated with linsitinib and stimulated with CD3/CD38 and cells treated with linsitinib and stimulated with LPS (**Figure 8C**). The uptake of [^3^H] arginine was measured over 24 h. The results reveal no differences between the uptake of arginine between either group, respectively (**Figure 8C**). These findings demonstrate, that the depletion of arginine is in indeed attributed to an upregulation of arginase and not an inhibition of arginine uptake, even in a state of immune activation, which was mimicked by CD3/CD28 and LPS.

## 4 Discussion

Here, we demonstrate that immunization against TSHR A-subunit plasmid results in influx of T lymphocytes into the bone marrow, activation of the bone marrow as indicated by stem cell proliferation, release of pro-inflammatory cytokines and a reduction of anti-inflammatory cytokines. All of the observed changes in the bone marrow were prevented by linsitinib. Linsitinib mediates, at least in part, immune inhibition in the bone marrow by maintaining a normal level or by even upregulation of arginase-1, which consumes and thereby down-regulates arginine. Low arginine levels are known to prevent activation of T-lymphocytes (44, 45). Reconstitution of arginine overcomes the inhibitory effect of linsitinib on bone marrow cells. Our studies identify several novel mechanisms: First, we demonstrate that the bone marrow is activated in autoimmune GD and based on previous data on EAE (19), it is very likely that this activation is an important component for the development of TED. Second, in a previous study, we have demonstrated that linsitinib prevents development of thyroid eye disease after TSHR immunization (18). Here, we uncover novel molecular mechanisms how the IGF-1R and IR inhibitor linsitinib mediates immune inhibition by upregulation of arginase-1 in the bone marrow and subsequent release of IL-10 and PGE_2_.

This is the 1^st^ report showing a potential role of the bone marrow in TED. Thus, the present concepts need to be confirmed in further mouse and human studies. Future studies are also required to define potential further effects of linsitinib on the bone marrow of immunized and non-immunized mice and, in addition, these studies should be also transferred to humans.

At present, it is unknown how inhibition of IGF-1R by linsitinib mediates upregulation of arginase-1 in the context of an autoimmune disorder in the bone marrow. However, it has been shown that inhibition of mTOR by rapamycin (Sirolimus) induces formation of myeloid-derived suppressor cells (46, 47). Inhibition of mTOR in myeloid-derived suppressor cells resulted in an upregulation of arginase-1 expression, which suppressed T-cell proliferation (46, 47). A similar mechanism may apply to the effect of linsitinib, since AKT/mTOR is an important downstream pathway for IGF-signaling and might be inhibited by linstinib. This is consistent with a recently published study on treatment of active TED with rapamycin which significantly improved the disease outcome and inflammation in comparison to first line therapies, such as intravenous methylprednisolone (48).

Thus, linsitinib might upregulate arginase-1 expression by blocking important downstream signaling pathways of the IGF-1R, such as mTOR.

Arginase-1 has been shown to be involved in the development of diabetes and it has been shown to be involved in insulin resistance by several mechanisms, including alterations of the balance between M1 and M2 macrophages and alteration of macrophage infiltration into fat tissue. It might be possible that arginase expression in the bone marrow induces a similar shift of macrophages to a phenotype corresponding to myeloid-derived suppressor cells and therefore contribute to immune suppression by linsitinib.

Our studies suggest an important function of arginase-1 in the pathogenesis of GD and TED, a pathway which is targeted by linsitinib. Arginase-1 is a metalloenzyme that catalyzes the conversion of L-arginine to L-ornithine and thus is a key player in the urea cycle (39). The enzyme exists as two isoforms, Arginase-1 which is located in the cytoplasm expressed at high levels in the liver and Arginase-2 which is located in the mitochondria and expressed in different tissues, e.g. at high levels in the kidney (39, 49). Myeloid cells, like macrophages, myeloid-derived suppressor cells (MDSC) or granulocytes metabolize arginine via arginase-1, but also other immune cells such as regulatory T-cells express arginase-1 in order to metabolize arginine (42, 50, 51). Arginase-1 is able to modulate T-cell function through the availability of the semi-essential amino acid arginine, which is essential for T-cells for survival, proliferation, differentiation, cytokine production and effector functions (44, 45, 52). It has been shown, that T-cells rely on arginine for various key biological processes such as memory function, expression of the T-cell receptor and the CD3ζ and therefore, arginine depletion through an upregulation of Arginase leads to an impaired T-cell function (42, 51, 53, 54). Stimulation of autoreactive T-cells is involved in triggering the autoimmune response in GD (55) and these autoreactive T-cells very likely rely on arginine in order to become activated, to proliferate and to drive the disease formation and progression. Depletion of arginine through an upregulation of arginase-1 in the bone marrow upon treatment with linsitinib will therefore result in downregulation of T-cell functions and attenuate the autoimmune response.

In the present study we suggest arginase-1 as a key player in modulating the autoimmune response in the bone marrow, triggered by immunization of mice with the TSHR in order to induce GD and consecutive thyroid eye disease. Mice immunized with the TSHR showed an inhibition of arginase-1, which leads to higher arginine concentrations within the bone marrow, which then allows immune cells like T-lymphocytes to proliferate and mediate the disease onset. In addition, the anti-inflammatory cytokine IL-10 and PGE_2_ were down-regulated depending on arginase-1 inhibition, as evidenced in the reconstitution experiments, further escalating the autoimmune reaction. Linsitinib reversed these events by an upregulation of arginase-1 expression, depletion of arginine and upregulation/maintaining of IL-10 and PGE_2_, events that collectively prevent and inhibit the autoimmune response. IL-10, as an anti-inflammatory cytokine has also been shown to induce arginase-1 expression (42). As linsitinib also induces the production of IL-10 in an arginine dependent manner, the upregulation of arginase might lead to IL-10 production, which then further activates arginase-1 in a positive feedback loop, amplifying the immune inhibitory response. In an anti-inflammatory micromilieu induced by IL-10 production, macrophages drive towards the M2-type, which is also known to prevent inflammation, in contrast to the pro-inflammatory M1-type (42).

Osteopontin has been shown to be important in inflammatory processes, by influencing and stimulating various immune cells such as macrophages, neutrophils, natural killer cells, T-cells, B-cells and dendritic cells (34). It has been shown in multiple sclerosis, that osteopontin actively exacerbates the clinical course of the disease in a mouse model and in addition is elevated in relapses of multiple sclerosis patients (56). Moreover, osteopontin and its receptor CD44 are also elevated in other autoimmune disorders, such as experimental colitis, an animal model of human inflammatory bowel disease (57). Importantly, osteopontin has been suggested to be relevant in TED (7, 58). Recent studies revealed an increase of osteopontin and CD44 in patients with GD and the mRNA and protein levels of osteopontin showed an increase in orbital tissue from patients suffering from active thyroid eye disease (58). Osteopontin stimulated orbital, fibroblasts, which are key players in the local pathogenesis of thyroid eye disease (7) to proliferate and migrate through the PI3K/Akt signaling pathway (58). However, the role of osteopontin in the bone marrow for pathogenesis of thyroid eye disease is presently unknown. We would interpret the release of osteopontin and IL-6 as mechanisms for activation of the innate immune system, which is required for stimulation of T-lymphocytes in the bone marrow, similar to the findings of T cell stimulation in the bone marrow during the induction of EAE (19). The inhibitory effects of linsitinib on IL-6 and osteopontin formation might be indirectly mediated by upregulation of myeloid-derived suppressor cells via arginase-1 expression or directly mediated by interfering with pathways such as PI3K-activation that are known to regulate osteopontin and IL-6 expression (57, 59, 60)

Our findings are in line with previous studies in an EAE model that demonstrated a central role of the bone marrow in stimulation of autoreactive T-lymphocytes before they migrate into the brain (19). However, the studies focused on molecular mechanisms mediating the interaction of the bone marrow with T-lymphocytes, while the present study focuses on the lack of control of immune-inhibitory mechanisms in the bone marrow during an auto-immune disorder and the reconstitution of these mechanisms by linsitinib.

A release of osteopontin in the bone marrow and/or the formation of osteopontin-producing immune cells might also affect orbital cells, in particular orbital fibroblasts. Osteopontin has been shown to interact with integrins and CD44 receptors and recruit inflammatory cells (34). Thus, a potential axis between the bone marrow and the orbit might contribute to the local inflammation in the orbit, but this needs to be investigated in future studies.

An increase of body weight might further promote the formation of osteopontin in fat tissues and thereby contribute to inflammation. However, in our experiments we did not observe a significant increase of body weight, neither in the immunized group nor in the immunized + linsitinib-treated group suggesting that the observed formation of osteopontin is independent of fat tissue activation upon immunization.

In the experiments employing i.p. injections of linsitinib into C57BL/6 mice, we aimed to perform an independent analysis of the effects of linsitinib on the bone marrow, but we also wanted (i) to confirm the key findings of our studies obtained on immunized BABLB/c-mice in another mouse strain to exclude that the effects of linsitinib are restricted to a specific strain and (ii) we aimed to show that the mode of application (gavage vs i.p.) does not alter the effects of linsitinib on arginase expression.

In addition, i.p. injection of linsitinib highly ensures that the drug (linsitinib) is absorbed, which is very useful as we also only injected the mice 5 times. On the contrary, this could not be performed with the initial linsitinib treatment via gavage, as i.p. injections for such a long time frame significantly increase the risk for infection.

The studies confirmed that neither the mouse strain nor the application method alters the effects of linsitinib on arginase expression.

Several studies have shown a crosstalk between the TSHR and IGF-1R, mainly in fibroblasts (12, 15, 61, 62). Involvement of the Insulin receptor (IR) in the disease pathogenesis of Graves’ Disease and thyroid eye disease has, to our knowledge, not been reported yet. However, it might be possible that IGF-1R and IR also interact in immune cells of the bone marrow and thereby linsitinib also interferes signaling of the IR, although this has not been investigated. Further, linsitinib might down-regulate intracellular signaling pathways such as the Erk and Akt signaling pathways and this might (indirectly) interfere with IR signaling in bone marrow cells. However, it is unknown whether these events occur in bone marrow cells and these hypotheses require further studies.

Hyperglycemia is a potential adverse event of linsitinib, which we also investigated in our study by measuring the blood glucose levels of the mice (18). However, we could not detect a trend towards elevated blood glucose levels in mice treated with linsitinib suggesting that under the present conditions linsitinib primarily acts via the IGF-1R.

Our findings indicate that linsitinib leads to an upregulation of arginase-1, which decreases the availability of arginine for T-cells and thus inhibits the T-cell activation and inflammatory response. Thus, drugs that stimulate arginase-1 or increase expression of arginase-1 might be beneficial in thyroid eye disease and linsitinib is one example for such a novel drug. Moreover, treatment with arginase-1 or arginase-1 activators/inducers might potentiate the effect of linsitinib, making it an even more efficient drug. However, it is important to find the right balance between the immunosuppressive effect on the bone marrow, which is wanted and needed in order to treat autoimmune disorders, and complete immunosuppression allowing, for instance infections.

In addition, arginase-1 could serve as a prognostic biomarker. Patients with high levels of arginase-1 in the blood or bone marrow should have a milder course of the disease then patients with low levels of arginase-1. However, these are just speculations which need to be tested in clinical studies.

The data are the first report of a role of the bone marrow in the pathophysiology of TED. However, they have not been confirmed by performing the same experiments on an 2nd independent mouse cohort and, therefore, should be considered in this respect as preliminary until independent confirmation. The method of immunization and induction of the autoimmune condition Graves’ Disease has been independently repeated by several studies from different scientist, also several times from our lab in which we identified the mouse model as robust and reproducible (25, 63–65).

## 5 Conclusion

In summary, we investigated the development of Graves’ Disease and thyroid eye disease in a murine mouse model after immunization with the A-subunit of the TSHR. We suggest the bone marrow as a new key player to trigger the autoinflammatory response of the disease and to promote disease development of thyroid eye disease. The oral, small molecule IGF-1R and IR antagonist linsitinib blocks the activation of the bone marrow and thus inhibits disease progression and development of TED. Linsitinib reduces proinflammatory cytokines and inflammation in the bone marrow and inhibits T-cell activation by upregulation of the enzyme arginase-1. This local immunosuppressive effect of linsitinib and the potential importance of the bone marrow in TED development gives insight into a new understanding of the disease and potential new therapeutic options to treat TED which implicates the clinical significance of our findings.

## Data availability statement

The original contributions presented in the study are included in the article/supplementary materials, further inquiries can be directed to the corresponding author.

## Ethics statement

The animal study was approved by the North Rhine Westphalian State Agency for Nature, Environment and Consumer Protection (LANUV), Germany, or by the local IACUC, University of Cincinnati, Cincinnati, USA. All experiments were performed according to the FELASA regulations and ARRIVE guidelines. The study was conducted in accordance with the local legislation and institutional requirements.

## Author contributions

AG: Study design, data collection and analysis, performing of statistical analysis, writing and preparing of the original draft, project administration; MH: helped with data collection, SK: helped with the ELISA, MS: performed bone marrow sections, BW: helped with the ELISA, GW: Resources, Reviewing of the manuscript, RZ: Funding Source, resources, GH: Resources, AD: helped with data collection, NB: Resources, AE: Resources, supervision, Reviewing and Editing of the manuscript, G-EG: helped with the data collection and analysis, resources, supervision, reviewing and Editing of the manuscript.
